# Characterization of Phosphorus in Animal Manures Collected from Three (Dairy, Swine, and Broiler) Farms in China

**DOI:** 10.1371/journal.pone.0102698

**Published:** 2014-07-22

**Authors:** Guohua Li, Haigang Li, Peter A. Leffelaar, Jianbo Shen, Fusuo Zhang

**Affiliations:** 1 Center for Resources, Environment and Food Security (CREFS), China Agricultural University, Beijing, China; 2 Plant Production Systems Group, Wageningen University, Wageningen, The Netherlands; Tennessee State University, United States of America

## Abstract

In order to identify the phosphorus species and concentration in animal manure, we comparatively characterized phosphorus in dairy manure, swine manure, and broiler litter, using a sequential procedure, a simplified two-step procedure (NaHCO_3_/NaOH+EDTA), and a solution Phosphorus-31 Nuclear Magnetic Resonance (^31^P-NMR) spectroscopy procedure. In the sequential procedure, deionized water extracted 39, 22, and 32%; NaHCO_3_ extracted 48, 26, and 37%; NaOH extracted 8, 9, and 13.8%; and HCl extracted 3, 42.8, and 17% of the total phosphorus in dairy manure, swine manure and broiler litter, respectively. Total phosphorus extracted by the NaHCO_3_/NaOH+EDTA procedure was 7.5, 32.4, and 15.8 g P kg^−1^ for dairy manure, swine manure, and broiler litter, respectively. The solution ^31^P-NMR procedure detected that 9, 34, and 29% of total phosphorus was phytic acid in dairy manure, swine manure, and broiler litter, respectively. These results show that phosphorus forms, availability, and quantities differ between animal manures, which provides valuable information for P characterization of animal manures in China.

## Introduction

Due to the rapid economic development of China since 1980, the diet of residents has shifted from predominantly plant to more animal products [1]. Livestock and poultry breeding have increased considerably by the stimulation of consumer demand for animal products, and consequently a vast expansion in animal production has occurred. Modes of animal production have shifted gradually from conventional family-based animal rearing to intensive animal feeding operations, with more input of protein and energy into animal feeds. These differences may significantly impact forms, bioavailability, utilization, and cycling of manure nutrients and their potential impact on soil and water quality. For example, if diets of dairy cattle generally exceed animal phosphorus (P) requirements by 25% to 40%, a significant fraction (∼80%) of the P consumed is excreted in the manure due to the low utilization efficiency of dietary P [2].

The excess of total P in animal feed is common throughout China, which has led to the production of high-concentration of animal manure P [3]. The capacity of soil to hold manure P mainly depends on the forms and bioavailability of P in manure [4]. If long-term manure application to the arable land is practiced without concerning the P forms and bioavailability, it may lead to soil P accumulation and an acceleration of eutrophication [5]. However, little detailed information about the relative quantities and forms of P in animal manure in China is available to farmers now. Thus, characterizing manure P becomes critical to better understand manure P dynamics and assist farmers in making optimal management of manure P for reducing water pollution.

Traditionally, the composition of animal manure P has been analyzed by three solution-based techniques, including sequential fractionation [6]–[10], enzymatic hydrolysis [11]–[15] and solution ^31^P-NMR spectroscopy [16]–[18]. The sequential procedure of Hedley et al. (1982) [19] originally developed for soil P analysis was adapted for characterizing P in three contrasting manures (broiler litter, dairy manure, swine manure) for the relative dissolution and fraction distribution with deionized water, 0.5 *M* NaHCO_3_, 0.1 *M* NaOH, and 1.0 *M* HCl and concentrated sulfuric acid. Generally, H_2_O and NaHCO_3_ primarily extract labile P (i.e., plant-available) and NaOH primarily extracts organic P; HCl was assumed to extract inorganic forms of P, such as relatively stable Ca-bound phosphate. For example, Dou et al. (2000) [7] reported that deionized water, NaHCO_3_, NaOH and HCl extracted 70, 14, 6, and 5% of the total P in the dairy manure, and 49, 19, 5, and 25% of the total P in the poultry manure sample, respectively. Ajiboye et al. (2004) [20] found the majority of P in fresh dairy, beef, and swine manure was in labile P pools, which can be extracted by NaHCO_3_ and NaOH. In order to further understand the structural information of manure P, solution ^31^ P-NMR was used to quantify P concentrations and compounds present in a simplified two-step fractionation procedure which involved extraction of readily soluble phosphorus in 0.5 *M* NaHCO_3_ followed by extraction of stable phosphorus in a solution containing 0.5 *M* NaOH and 50 m*M* EDTA [13], [17], [21], [22]. The solution of NaOH-EDTA has a greater power to extract stable metal-P and organic-P species. Turner (2004) [17] used ^31^P-NMR to identify phytic acid in alkaline extracts of broiler litter and swine manure, while Leinweber et al. (1997a) [6] identified broad classes of P species, including orthophosphate monoesters and diesters, and pyrophosphates in swine and poultry manure. Information on the forms of phosphorus contained in animal manures is a pivotal factor determining its environmental fate [23].

The objective of this study was to identify the P species and concentration in dairy manure, swine manure, and broiler litter collected from three farms in China using three different methods, including sequential fractionation, NaHCO_3_/NaOH+EDTA extraction procedure and solution ^31^P-NMR, respectively.

## Materials and Methods

### Sample collection

Three types of manure were collected from private livestock farms in China which did not involve endangered or protected species: swine manure (the swine was grain fed in housing system) from Beilangzhong farm, located at Zhaoquanying town, Shunyi district, Beijing (40°10′ N, 116°35′ E), Xuejun Liu was responsible to future permissions; dairy manure (the dairy was pasture fed in sheds) from an individual farm located at Shandong Agricultural University Experimental Station, Taishan district, Taian, Shandong Province (36°9′ N, 117°9′ E), Zhiguang Liu was responsible to future permissions; and broiler litter (the broiler chicken was grain fed in coop system) from an individual farm located at Xizhengqiao village, Jining, Shandong Province (35°20′ N, 116°31′ E), Dianting Li was responsible to future permissions. These three farms were all private farm. We had gotten the farmer's permission before collecting animal manure from their farms for P species analysis. The collected manure was stored immediately in a portable refrigerator, which could avoid the potential damage to public health. So there was no need to be approved by an Institutional Animal Care and Use Committee (IACUC) or equivalent animal ethics committee. For each type of manure, multiple samples were collected from animals at the same growth stage from different locations in the facility, then composited and subsampled. Animal feed samples were also collected 24 h before manure sampling to ensure that sampled feed represented the true source of manure P. Manure samples were stored below 4°C and analyzed within a week. Manure samples were dried at 65°C, ground to <2 mm, and stored in sealed containers at room temperature for the sequential extraction procedure. Manure pH was determined on a 10:1 water (mL) to dry matter ratio (g). Total elements in samples were determined by microwave digestion in concentrated HNO_3_ and H_2_O_2_ (*EPA Method 3052*) with detection by inductively coupled plasma-optical-emission spectrometry (ICP-OES) (Table 1). The concentration of total P in feeds was analyzed after the samples were digested with concentrated H_2_SO_4_-HNO_3_ for 75 min at 350°C [24], using the phosphomolybdate blue method [24].

**Table 1:** Selected characteristics of the animal manures used in this study.

Manure	pH†	Al‡	Ca	Fe	Mg	Mn	P	C	N	C/N Ratio§
		g kg^−1^ dry weight						mg kg^−1^		
Dairy manure	8.3	0.97	17.4	1.3	11	0.2	7	39	2	20
Swine manure	8.2	0.87	51.1	1.1	10	0.4	32	37	3	12
Broiler litter	6.4	0.59	24.2	0.7	7.6	0.2	14	36	5	7

**Note:**

† pH was determined on a 10:1 water (mL) to dry matter ratio (g).

‡ Total elements in three animal manures determined by microwave digestion in concentrated HNO_3_ and H_2_O_2_ and detection by ICP-OES

§ Total nitrogen and carbon in three animal manures determined by Vario MACRO CN.

### Sequential Fractionation

Manure samples were sequentially extracted by a modified procedure developed by Dou et al. (2000) [7] (commonly applied to manure P), which was based on Hedley et al. (1982) [19] for fractionation of soil P. In the sequential fractionation, manure samples were extracted sequentially with deionized water, 0.5* M* NaHCO_3_, 0.1* M* NaOH, 1.0 *M* HCl and concentrated H_2_SO_4_-HNO_3_. Each extraction was performed in a 1∶100 manure-to-solution ratio for 1 h [7]. For all procedures, extracts were made using 0.3 g dried, ground manure samples, shaken with 30 mL extractant at 20°C on a shaker at a speed of 150 excursions min^−1^ for one hour, centrifuged at 10000×g for 10 min, and then suction-filtered through 0.45 µm nitrocellulose membranes. The filtrate was analyzed for inorganic P (P_i_) and total P (P_t_). Organic P in the extracts was determined by difference of orthophosphate detected using the phosphomolybdate blue method of Murphy and Riley (1962) [25] before and after an aliquot filtrate was digested by sulfuric acid-persulfate [26], [27]. At the end of the shaking/filtering cycle, residues on the filter membrane and the screw-cap centrifuge tube were transferred to a digestion tube using deionized water. Manure P in residues was determined by digestion by refluxing with concentrated H_2_SO_4_ and HNO_3_ for 75 min at 350°C [24].

### NaHCO_3_/NaOH+EDTA procedure and solution ^31^P-NMR

The NaHCO_3_/NaOH+EDTA procedure involved initial extraction of manure in 0.5* M* NaHCO_3_ for 4 h, followed by extraction overnight (16 h) in a solution containing 0.5 *M* NaOH and 50 m*M* EDTA, which was first developed by Turner and Leytem (2004) [10]. Readily soluble P was extracted by NaHCO_3_ followed by extraction of stable P in a NaOH+EDTA solution. In the procedure, the manure-to-solution ratio was 1∶60 for the NaHCO_3_ and 1∶20 for the NaOH-EDTA extract [17]. Extractions were performed in the same way as the sequential fractionation. An aliquot (5 mL) of each extract was diluted and analyzed separately for P_i_ and P_t_. The remainder of the solution of replicate extract was then frozen rapidly at −80°C, lyophilized, and ground to a fine powder. Before ICP-OES analysis and lyophilization, the solutions extracted by NaHCO_3_ were acidified with dilute HCl to approximately pH 3 to dissolve carbonates [10]. Approximately 100 mg of each lyophilized extract was subsequently dissolved in 0.9 mL of a solution containing 1 *M* NaOH and 0.1 *M* EDTA, and 0.1 mL deuterium oxide (D_2_O) was added for signal lock before NMR spectroscopy. The inclusion of NaOH ensures consistent chemical shift at pH>13. Samples were then transferred to a 5-mm NMR tube and stored at 4°C before analysis within 12 h. Solution ^31^P-NMR spectra were obtained using a Bruker Avance DRX 400 MHz spectrometer (Beijing, China) operating at 202.456 MHz for ^31^P and 500.134 MHz for ^1^H [10].

P compounds from the ^31^P-NMR spectra were identified by their chemical shifts of signals (ppm) relative to 85% H_3_PO_4_ [10], [17], [28], [29]. Spectra were measured using Mestre-C software. The orthophosphate peak in each spectrum was factitiously standardized to 6 ppm to simplify comparisons among spectra. Chemical shift of signals was used to identify P compounds, including orthophosphate (6 ppm), orthophosphate monoesters between 5.8 and 3.94 ppm, pyrophosphate (−4.53 ppm) and orthophosphate diesters between −0.31 and 3.91 ppm [10], [30], [31], [32]. The concentration of P compounds in extracts was calculated by multiplying the proportion of the spectral area assigned to the corresponding signal by the total phosphorus concentration (mg P kg^−1^ dry manure) in the original extract.

## Results and Discussion

### Total P concentration of animal manures

Total P concentration of animal manures determined by ICP ranged from 32 g P kg^−1^ in swine manure, 14 g P kg^−1^ in broiler litter and 7 g P kg^−1^ in dairy manure. Dou et al. (2003) [33] reported a total P concentration rang of 6 to 12 g P kg^−1^ in dairy manures from 70 farms in USA. Turner and Leytem (2004) [10] reported a total P concentration of 15.9, 4.9, and 14.6 g P kg^−1^ in broiler litter, cattle manure and swine manure in USA, respectively. Maguire et al. (2004) [34] reported a concentration range of 8.6 to 17.8 g P kg^−1^ in broiler litter in USA. Pagliari and Laboski (2012) [35] reported that total P recovered in the sequential fractionation averaged 7.4, 6.5, 19.4, 21.2, and 33.9 g P kg^−1^ in beef, dairy, turkey, chicken, and swine manure, respectively, collected from private farms and the University of Wisconsin Agricultural Research Station in Arlington, Wisconsin. Generally, the results in this study indicate a similar total P concentration for dairy manure and broiler litter, but a greater of total P concentration for swine manure in China compared with developed countries and much higher than those in China twenty years ago, which was only 9.0 g P kg^−1^ [36]. The difference in swine manure is likely to be caused by mineral P supplementation of the swines' diet [8]. The concentration of total P in swine feed is much higher than the feed standard which leads to the high-concentration of P in excreted manure (Table 2) [37]. In this study, the concentration of total P in swine manure was 4.4 times greater than that in dairy manure and 2.3 times that in broiler litter. So, more attention needs to be paid to the long-term swine manure application to the agricultural land which often leads to soil P accumulation above the amounts sufficient for optimal crop yields and an increased potential risk for P loss in runoff as well as in leachate [5]. Total Al, Fe, Mg, and Mn concentrations were greater in dairy manure and swine manure than in broiler litter, which may be the result of high pH in swine and dairy manure (Table 1). The concentration of Ca was significantly higher in swine manure than that in the other two types of manure due to the Ca-P additives in the diet. Organic C was similar among all three types of manure, whereas broiler litter contained the greatest amount of nitrogen (Table 1).

**Table 2:** The concentration of total P in animal feed.

Item	Dairy feed	Swine feed	Broiler feed
		g P kg^−1^	
Total P†	3.2	7.3	4.9

**Note:**

† Total phosphorus was determined using H_2_SO_4_-H_2_O_2_ digestion of original animal feed, and then tested using Murphy-Riley (1962).

### Sequential fractionation

In the sequential fractionation procedure, the percent of cumulative P in dairy manure was 39 (e.g., 39 is the sum of inorganic and organic P), 48, 8, 3 and 2% associated with extractants H_2_O, NaHCO_3_, NaOH, HCl and residues, respectively (Table 3). Corresponding P fractions for the swine manure and broiler litter, H_2_O extracted 22 and 32% (as H_2_O-P); NaHCO_3_-P, 26 and 37%; NaOH-P, 9 and 13.8%; HCl-P, 42.8 and 17%; and residue-P, 0.2 and 0.2% of the total P, respectively (Table 3). In these three types of manure, more P was in the NaHCO_3_ and H_2_O extracted fractions followed by NaOH and HCl extracted fractions which suggested an increased risk of P loss through dissolving in rainwater or irrigation water from manure when surfaced applied. The majority of P components in dairy and swine manure were inorganic (68% and 58%), while the concentration of inorganic- and organic P was about equal in broiler litter. The concentration of P extracted by HCl from swine manure was higher than that from other manures. It is probable that Ca-P is the primary complex because Ca^2+^ is the dominating cation in swine manure [38], [39], [40], [41]. He et al. (2006, 2008) [12], [14] found some organic P presented in the HCl extracted fraction, which was also confirmed by Takahashi (2013) [42]. In this study, P_o_-HCl was not determined that may cause underestimating of total organic P. The proportion of organic P to total P in dairy manure was 30%, which was lower than swine manure (41.8%) and broiler litter (49.8%) (Table 3). The reason may be the dairy manure was from ruminant animal, which could secrete phytases and other phosphatases from their gut to increase the organic P hydrolysis [34], [43]. Another reason may be that there was less phytate in the diet for dairy, particularly when the dairy was grazed on grassland.

**Table 3:** The concentration of phosphorus compounds in sequential extracts of animal manures from the modified Hedley fraction procedure determined by Murphy-Riley (1962).

P fraction†	Dairy manure	Swine manure	Broiler litter
		g P kg^−1^ of dry matter	
	Inorganic P		
H_2_O-P_i_	2.1 (31%)‡	5.0 (16%)	3.2 (29%)
NaHCO_3_-P_i_	2.4 (35%)	6.5 (21%)	1.3 (12%)
NaOH-P_i_	0.07 (1%)	0.8 (3%)	0.3 (3%)
HCl-P_i_	0.07 (1%)	5.7 (18%)	0.7 (6%)
∑ P_i_ fractions	4.64 (68%)	18.0 (58%)	5.5 (50%)
	Organic P		
H_2_O-P_o_	0.5 (8%)	2.0 (6%)	0.3 (3%)
NaHCO_3_-P_o_	0.89 (13%)	1.6 (5%)	2.8 (25%)
NaOH-P_o_	0.48 (7%)	1.9 (6%)	1.2 (10.8%)
HCl-P_o_	0.15 (2%)	7.8 (24.8%)	1.3 (11%)
∑ P_o_ fractions	2.0 (30%)	13.3 (41.8%)	5.6 (49.8%)
Residual P¶	0.15 (2%)	0.09 (0.2%)	0.02 (0.2%)
Total P	6.79	31.4	11.1

**Note:**

† H_2_O-P_i_ and H_2_O-P_o_ are water-extractable inorganic and organic phosphorus; NaHCO_3_-P_i_ and NaHCO_3_-P_o_ are NaHCO_3_-extractable inorganic and organic phosphorus; NaOH-P_i_ and NaOH-P_o_ are NaOH-extractable inorganic and organic phosphorus; HCl-P_i_ and HCl-P_o_ are HCl-extractable inorganic and organic phosphorus.

‡ Inorganic phosphorus (P_i_) in each extract was determined by Murphy-Riley (1962). Organic phosphorus (P_o_) in each extract was calculated as the difference between P_t_ and P_i_. Values in parenthesis are the percentage of total manure phosphorus in each extract.

¶ Residual P was determined using H_2_SO_4_-H_2_O_2_ digestion of the residual.

### NaHCO_3_/EDTA procedure

Total P extracted by the NaHCO_3_/EDTA was 7.5, 32.4 and 15.8 g P kg^−1^ in dairy manure, swine manure and broiler litter, respectively (Table 4). The recovery ratio of total P from manures was from 101% to 109% which confirmed that the procedure was an effective method to extract P from these manures (Table 4). The P extracted in this procedure was higher than that obtained with the sequential procedure. The probable reason was the P losses during the filtration through the 0.45 µm nitrocellulose membranes in the sequential procedure. For the readily soluble P fraction, an initial NaHCO_3_ extraction recovered less total P from three manures than the combined deionized water plus NaHCO_3_ extraction in the sequential procedure (Table 4). This may be due to the presence of a high concentration of phosphate ions contained in the animal manure, while an initial NaHCO_3_ extraction could not supply sufficient carbonate ions to exchange the phosphate ions absorbed on the surface of the particles. Another reason may be that two extractions are more powerful than one extraction because of a dilution effect. We also noted that about 15–19% of total P was left in a residual P fraction, compared with that in the sequential procedure. It is probable that relatively stable Ca-bound phosphate, which was the major binding complex, could not be extracted completely by NaOH+EDTA in the procedure [39]. However, the alkaline solution improved organic P recovery from animal manure and facilitated the spectral resolution for subsequent ^31^P-NMR analysis [10], [17], [18].

**Table 4:** Concentration of phosphorus compounds in two steps extracts of animal manures determined by Murphy-Riley (1962).

P fraction†	Dairy manure	Swine manure	Broiler litter
		g P kg^−1^ of dry matter	
	Inorganic P		
NaHCO_3_-P_i_	3.6 (48%)	4.6 (14%)	3.5 (22%)
NaOH/EDTA-P_i_	0.3 (4%)	2.3 (7%)	2.8 (18%)
∑ P_i_ fractions	3.9 (52%)	6.9 (21%)	6.3 (40%)
	Organic P		
NaHCO_3_-P_o_	0.3 (4%)	0.3 (1%)	1.4 (9%)
NaOH/EDTA-P_o_	2.2 (29%)	19.0 (59%)	5.7 (36%)
∑ P_o_ fractions	2.5 (33%)	19.3 (60%)	7.1 (45%)
Residual P¶	1.1 (15%)	6.2 (19%)	2.4 (15%)
Total P	7.5	32.4	15.8

**Note:**

† NaHCO_3_-P_i_ and NaHCO_3_-P_o_ are NaHCO_3_-extractable inorganic and organic phosphorus; NaOH/EDTA-P_i_ and NaOH/EDTA-P_o_ are NaOH/EDTA-extractable inorganic and organic phosphorus.

‡ Inorganic phosphorus (P_i_) in each extract was determined by Murphy-Riley (1962). Organic phosphorus (P_o_) in each extract was calculated as the difference between P_t_ and P_i_. Values in parenthesis are the percentage of total manure phosphorus in each extract.

¶ Residual P was determined using H_2_SO_4_-H_2_O_2_ digestion of the residual.

### Solution ^31^P-NMR spectra

**Figure 1:**
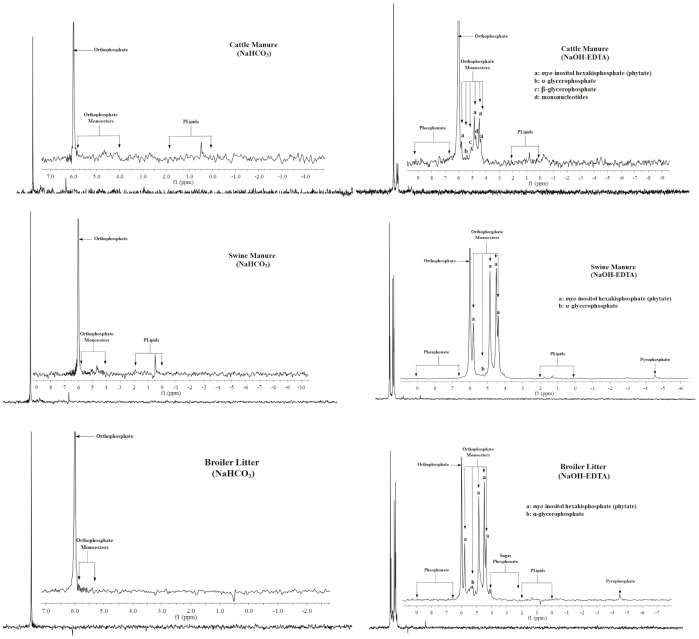
Solution ^31^P-nuclear magnetic resonance spectra of sequential extracts of dairy manure, swine manure, and broiler litter involving extraction in 0.5 *M* NaHCO_3_ and a solution of 0.5 *M* NaOH and 50 m*M* EDTA. The main spectra were scaled to the full height of the phosphate signal at approximately 6.0-6 to 9 ppm.

Solution ^31^P-NMR spectra of two extracts from three types of animal manure in the NaHCO_3_/EDTA procedure were shown in Figure 1. An orthophosphate peak was identified in spectra from all extracts. However, peaks assigned to orthophosphate monoesters and diesters were much less and weaker in NaHCO_3_ extract spectra than the spectrum from NaOH-EDTA, which indicated the relatively lower concentration compared with those phosphates in the NaHCO_3_ extracts. The results were similar to those of Turner and Leytem (2004) [10] and He et al. (2009) [15]. In NaHCO_3_ extract spectra, P species, except orthophosphate, were almost undetectable at low resolution. However, P species identified in the NaOH-EDTA extract spectrum of broiler litter and swine manure were abundant (Figure. 1). All peaks presented in the spectrum from NaOH-EDTA were subsequently grouped into P species or P classes if specific identification could not be made, according to the literature (Table 5). Inorganic P species identified from the spectrum included orthophosphate at 6 ppm and pyrophosphate at −4.53 ppm. Detected organic P species included orthophosphate monoesters between 5.8 and 3.94 ppm, while orthophosphate diesters were between −0.31 and 1.32 ppm [29], [30], [32], [44]. The dominant P species in the NaOH-EDTA extracts was orthophosphate at 6 ppm which accounted for 13–27% of total P extracted from three different manures. The following most abundant of P species were phytate (mainly *myo*-inositol hexakisphosphate and α-glycerophosphate) at 5.8, 4.86, 4.49, and 4.38 ppm in the orthophosphate monoesters region (4.38 to 5.8 ppm) in all manures (Table 5). The total concentration of phytate (P species at 5.8, 4.86, 4.49, and 4.38 ppm) was 0.68, 11.29, and 4.61 g kg^−1^ accounting for 9, 34, and 29% of total P in dairy manure, swine manure and broiler litter, respectively. A similar result was also reported in Toor et al. (2005b) [45]. The possible explanation for this difference was that the ruminant animal of dairy cattle secretes phytase to digest phytate contained in feed which resulted in a reduced quantity of phytate in manure [34], [45], [46]. Some organic P species such as phytate were more strongly sorbed to soil particles while many others (e.g., orthophosphate diesters) were poorly absorbed to soil, which could lead to localized areas of soil P saturation and P loss [47]. To overcome the low digestibility of phytate P in nonruminant diets and decrease the total P content and organic P species, feed additives such as phytase and other enzymes are necessary, and thus could greatly reduce surpluses of manure P in areas of intensive animal production. For example, Maguire et al. (2004) [34] reported that phytase added to feed could decrease phytate P in broiler litter by 25 to 38%. Pyrophosphate was identified in swine manure and broiler litter, accounting for 0.5% of total P. The concentration of orthophosphate diesters detected in dairy manure and broiler litter has seldom been compared with monoesters which would include intact phospholipids.

**Table 5:** Functional classes of phosphorus compounds in a second extraction with a solution containing 0.5 *M* NaOH and 50 m*M* EDTA in the revised fractionation procedures detected by ^31^P-nuclear magnetic resonance spectroscopy (-0.31 to 6 represent the chemical shift, ppm; percentage of P forms of total P in parentheses).

Manure types	Inorganic P		Organic P										
	Orthophosphate	Pyrophosphate	Orthophosphate monoesters									Orthophosphate diesters	
	6	−4.53	5.8	5.63–5.10	5.07	4.86	4.75	4.49	4.38	4.25–3.94		1.32	−0.31
						g P kg^−1^ (% of P in extracts)							
Dairy manure	1.48 (20%)	ND	0.11 (1%)	ND	0.12 (2%)	0.23 (3%)	0.14 (2%)	0.21 (3%)	0.13 (2%)	ND		ND	0.074 (1%)
Swine manure	8.84 (27%)	0.15 (0.5%)	2.09 (6%)	0.92 (3%)	ND	3.7 (11%)	ND	3.6 (11%)	1.9 (6%)	ND		ND	ND
Broiler litter	2.02 (13%)	0.085 (0.5%)	0.79 (5%)	0.82 (5%)	0.11 (1%)	1.42 (9%)	0.55 (3%)	1.62 (10%)	0.78 (5%)	0.28 (2%)		0.027 (0.2%)	ND

**Note:**

ND: not detected.

-0.31-6: the chemical shift, ppm; percentage of P forms of total P in parentheses.

In general, The NaHCO_3_/NaOH-EDTA procedure was suitable to be used as a simple and efficient test method to estimate the magnitude of vulnerable P with environmental relevance in manures which could help farmers identify optimal management techniques and treatments to reduce potential P runoff on farms. Based on the accurate analysis of organic P in NaOH-EDTA extracts by ^31^P-NMR, orthophosphate monoesters, especially phytate, were identified as the dominant P species in swine manure and broiler litter. Thus, feeding management including the use of feed additives such as phytase and reducing over-feeding to minimize excessive dietary P could be a necessary and critical practice to reduce phytate and total P excretion, respectively, in manures to reduce the potential risk of P loss to the environment associated with surface application of manures. This would particularly be useful for areas where limited arable land was combined with intensive animal farming. In the future, we must not only promote the development of analytical methods to measure manure P to better understand the manure P dynamics and enhance our capability in management, but also we should be concerned about the changes of P forms, bioavailability, and potential for transport in runoff and animal manure interaction with soil.

## Conclusion

In this study, the average total P determined by two different methods was 31.9 g P kg^−1^ in swine manure, 13.5 g P kg^−1^ in broiler litter and 7.1 g P kg^−1^ in dairy manure collected from three farms, respectively, which was similar with developed countries, and much higher than concentrations reported in China in the 1990s (8.5–9.5 g P kg^−1^ in swine manure, 8.7–9.9 g P kg^−1^ in broiler litter and 4.1–4.5 g P kg^−1^ in dairy manure in 1990s) [36]. Our results show that more than 48% of total P in swine manure, 87% of total P in dairy manure and 69% of total P in broiler litter was water-soluble P (P_t_ extracted by Water and NaHCO_3_), which is easily lost into the environment. It is reported that 1582 Gg of animal P was presumably lost to the environment in 2006 in China, which is a big threat to water quality [3]. Therefore, an integrated management practice is necessary to reduce manure P losses at animal farm scale, including animal diet modification, livestock manure collection, storage and field application.
